# PCR could be a method of choice for identification of both pulmonary and extra-pulmonary tuberculosis

**DOI:** 10.1186/1756-0500-4-332

**Published:** 2011-09-08

**Authors:** Iram Amin, Muhammad Idrees, Zunaira Awan, Muhammad Shahid, Samia Afzal, Abrar Hussain

**Affiliations:** 1 Division of Molecular Virology & Molecular Diagnostics, National Centre of Excellence in Molecular Biology, University of the Punjab, Lahore-53500, Pakistan

## Abstract

**Background:**

Nucleic acid amplification assays including PCR have revolutionized the detection of *Mycobacterium tuberculosis *(MTB). Tuberculosis spread to almost every organ of the body and is characterized on the basis of localization of infection. Therefore, different types of body fluids and tissues can be used for the detection of MTB.

From 2004 to 2010 total 766 different types of smear negative samples from patients, clinically suspected for tuberculosis were received and investigated at Division of Molecular Diagnostics, University of the Punjab Lahore for the diagnosis of tuberculosis. *Mycobacterial *DNA was extracted followed by PCR amplification.

**Findings:**

A total of 356 (46.5%) samples were found positive by PCR for MTB. These included; serum (4.8%), blood (36.3%), urine (46.6%), cerebro spinal fluid (CSF) (42.1%), ascetic fluid (67.6%), pleural fluid (52%), pericardial fluid (30%), pus (38.6%), bone marrow (60%), sputum (38.8%) and bronchoalveolar lavage (BAL) (70%). Over all there was no significant difference in males and females neither in different age groups for the identification of MTB.

**Conclusion:**

We conclude that PCR is a useful and sensitive tool for the early diagnosis of MTB in variety of clinical samples.

## Background

Tuberculosis (TB) is one of the leading chronic infectious bacterial with a death toll of almost 3 million and more than 8 million new cases each year [1]. It is also an established fact that 95% of TB cases occur in underdeveloped countries [2] including Pakistan where 2,68,000 new cases and 64,000 deaths take place each year [3]. TB is caused by various species of mycobacteria among which *Mycobacterium tuberculosis *(MTB) is the most frequent [4] that is a slow growing facultative intracellular parasite [5].

Generally tuberculosis is diagnosed by conventional methods like sputum smear microscopy, chest radiographic findings and culture studies [6]. The existing methodologies remain ineffective due to limitations due to low mycobacterium levels and/or time consuming procedures [7]. Accurate and early diagnosis of TB is important for effective management and timely treatment. At present nucleic acid amplification based assays are the most suitable choice for the identification of MTB that has eliminated diagnostic problems with improved detection rates in smear negative samples with high degree of sensitivity and specificity [6-9] in both pulmonary and extra-pulmonary cases. Pulmonary and extra pulmonary TB types are different on the basis of location of the infection which could be inside or outside lungs respectively [10]. Pulmonary disease is believed to be more common but extra-pulmonary remains quite severe because of unapparent and nonspecific outcome [11].

The current study was designed to determine the clinical utility of MTB PCR test for the rapid diagnosis of both extra-pulmonary and pulmonary TB in a variety of clinical samples from Pakistan targeting the MTB-specific Insertion sequence 986/6110 (IS986/IS6110) that is present in multiple copies in MTB complex. It is well- known that TB spread to different organs and locations within the body such as brain, bones, abdomen, heart, lymphatic system, urinary tract and lungs. Therefore, we used blood, serum, CSF, Ascetic fluid, Urine, Sputum, Bronchoalveolar Lavage (BAL), Pleural fluid, pericardial fluid, Pus and Bone marrow as specimens for MTB detection through PCR method.

## Methods

### Patient's samples

Total 766 specimens from patients with a high clinical suspicion of pulmonary or extra-pulmonary tuberculosis were received at Division of Molecular Diagnostics CEMB, University of the Punjab from July 2004 to October 2010 for the detection of MTB including Serum (n = 41), Blood (n = 88), Urine (n = 225), Cerebro Spinal Fluid (CSF) (n = 76), Ascitic Fluid (n = 133), Pleural Fluid (n = 98), Pericardial Fluid (n = 10), pus (n = 44), Bone Marrow (n = 5), Sputum (n = 36) and Bronchoalveolar Lavage (BAL) (n = 10). All these samples were smearing negative. Pulmonary tuberculosis was defined as tuberculosis of lungs, pleura, and mediastinal lymph nodes whereas extra-pulmonary TB is disease outside these sites. In Pakistan tuberculosis is highly endemic. Several TB clinics and surveillance centers in Pakistan offer free diagnostic facility mostly based on smear examination will all the limitation of low sensitivities. None of these Centers has the modern molecular techniques for the diagnosis of TB. Ethical approval was not needed for the current study as all the samples from the subjects were received for clinical diagnosis from tertiary collection points and we had not disclosed any identification of the subjects.

### Sample processing and Heat inactivation

Immediately receiving, the samples were incubated at 80 C for 10 minutes to kill MTB. The sputum samples were digested and decontaminated with NALC-NaOH method. All the samples were concentrated by centrifugation at 5000 × g for 5 minutes. The pellet was used for DNA extraction.

### DNA Extraction

Total DNA was extracted from samples using commercially available kit (Gentra Purescrift, USA) according to the procedure given in the kit protocol for each type of sample. The DNA pellet was reconstituted in 20 ul distilled water and stored at minus 20°C if not used that time for PCR amplification.

### PCR Amplification

PCR amplification was performed as previously described by Eisenach and colleagues [12]. The assay detects a 123-bp region from the M. tuberculosis complex-specific insertion sequence IS6110. Briefly, PCR amplification was performed for the detection of TB using forward primer (5'-CCT GCG AGC GTA GGC GTC GGT-3') and reverse primer (5'-CTC CTC CAG CCC CCC CTT CCC-3'). The PCR reactions were performed in a final volume of 25 micro letter contained 10 mM Tris-HCl (pH 8.3), 50 mM KCl, 2.5 mM MgCl_2_, 0.01% gelatin, 0.2 mM (each) de-oxynucleoside triphosphate, 1 mM each of forward and reverse primers and 1 U of Taq DNA polymerase Enzyme (Gibco BRL Life Technologies USA). The thermalcycler (Applied Biosystem Inc., PCR system 2700) was programmed to initially incubate the samples for 2 min at 94°C, followed by 35 cycles consisting of 94°C for 45 seconds, 53°C for 45 seconds and 72°C for 1 minute. Final extension was given at 72°C for 10 minutes. Specific TB DNA detection for each sample was identified by specific 123-bp DNA bands on 2% agarose gel, stained with ethidium bromide, and evaluated under UV transilluminator. The estimation of the sizes of PCR products was done according to the migration pattern of a 100-bp DNA ladder (Gibco BRL Life Technologies USA).

To avoid cross-contamination between samples and contamination of reagents or samples with PCR-amplified products or positive controls all the procedures for sample preparation and PCR included measures were observed as described by Kwok and Higuchi [13]. DNA extraction, preparation of reaction mixture and PCR amplification were carried out in three separate laboratory rooms located far away from each other.

In all steps filtered tips were used to avoid carry over contamination. All the samples were run in duplicate.

### Statistical analysis

Statistical analysis was done using SPSS program for window. Statistical significance for comparisons of proportions was determined by the Fisher exact test.

## Results

In the current study, smear negative TB samples of total 766 clinically suspected tuberculosis patients were analyzed for *mycobacterial *DNA detection in different types of samples. Of these 437 (57.04%) were from male patients and 329 (42.95%) were from female patients. The mean (± SD) age was 45 ± 17 ranged from 5 years to 79 years Ninety four percent of the cases had suspected Extra-pulmonary TB and 6% were suspected for pulmonary TB. Of the 766 cases, 356 (46.5%) were found positive for TB by PCR method. Different types of respiratory and non respiratory samples showed different positivity rates as described in Table 1. It is observed that 70% of the BAL samples were found TB positive followed by 67.6% TB positive CSF samples while pericardial TB samples were least positive (30%).

**Table 1:** Positivity rates of MTB in different types of samples (n = 766)

**Sr. No**.	Type of Sample	Total Samples Received	Found Positive	Percentage
1	Serum	41	2	4.8%
2	Blood	88	32	36.3%
3	Urine	225	105	46.6%
4	CSF	76	32	42.1%
5	Ascitic Fluid	133	90	67.6%
6	Pleural Fluid	98	51	52%
7	Pericardial Fluid	10	3	30%
8	Pus	44	17	38.6%
9	Bone Marrow	5	3	60%
10	Sputum	36	14	38.8%
11	BAL	10	7	70%
12	**Total**	**766**	**356**	**46.4%**

MTB: *Mycobacterium tuberculosis*; CSF: cerebro spinal fluid; BAL: bronchoalveolar lavage;

Table 2 translates the positive mycobacterial DNA in different types of samples in males and females. Overall there was no significant difference of TB positivity in males (46.4%) and females (46.5%). Males showed higher positivity rates in blood, ascetic fluid, pericardial fluid, pus and BAL samples while females showed higher rates in urine, CSF, Pleural fluid, bone marrow and sputum samples. The positivity was seen significantly high (p < 0.05) in females compared to males in urine samples.

**Table 2:** Positivity rates of MTB in males and females (n = 766)

**Sr.No**.	Type of Sample	Total Samples Received	Males Positive/Total (%age)	Female Positive/Total (%age)	p-value
**1**	Serum	41	0/27	2/14 (14.2%)	0.1
**2**	Blood	88	18/41 (43.9%)	14/47 (29.7%)	0.2
**3**	Urine	225	53/134 (39.5%)	52/91 (57.1%)	0.07
**4**	CSF	76	16/41 (39%)	16/35 (45.7%)	0.1
**5**	Ascitic Fluid	133	61/81 (75%)	29/52 (55.7%)	0.1
**6**	Pleural Fluid	98	30/58 (51.7%)	21/40 (52.5%)	0.5
**7**	Pericardial Fluid	10	3/9 (33.3%)	0/1	0.7
**8**	Pus	44	11/25 (44%)	6/19 (31.5%)	0.39
**9**	Bone Marrow	5	1/2 (50%)	2/3 (66.6%)	0.5
**10**	Sputum	36	5/13 (38.4%)	9/23 (39.1%)	0.2
**11**	BAL	10	5/6 (83.3%)	2/4 (50%)	0.5
**12**	**Total**	**766**	**203/437 (46.4%)**	**153/329 (46.5%)**	**NS**

MTB: *Mycobacterium tuberculosis*; CSF: cerebro spinal fluid; BAL: bronchoalveolar lavage; NS: Non-significant

Effect of age on samples positivity was also being evaluated. It was found that most of the positive samples lie in the age group of 41 to 60 years (35.6%) while 34.2% samples were positive in the age group of 21-40 years (Table 3).

**Table 3:** Positivity of MTB in different age groups (n = 356)

**Sr. No**.	Types of Samples	Found Positive	< 20 years	21-40 years	41-60 years	> 60 years	Unknown age
1	Serum	2	0	1	1	0	0
2	Blood	32	7	12	9	4	0
3	Urine	105	13	43	40	9	0
4	CSF	32	7	13	6	5	1
5	Ascitic Fluid	90	3	19	45	14	9
6	Pleural Fluid	51	1	17	17	13	3
7	Pericardial Fluid	3	0	3	0	0	0
8	Pus	17	4	10	2	0	1
9	Bone Marrow	3	1	1	1	0	0
10	Sputum	14	3	3	2	5	1
11	BAL	7	0	0	4	3	0
12	Total	356	39	122	127	53	15

MTB: *Mycobacterium tuberculosis*; CSF: cerebro spinal fluid; BAL: bronchoalveolar lavage;

## Discussion

Tuberculosis (TB) kills more people in Southeast Asia including Pakistan than any other infectious disease. According to World Health Organization (WHO) reports [1,14] Pakistan is among the 22 countries having a high incidence of TB where an increase in the rate of MDR-TB and XDR TB strains has been reported in this region of the world [15]. The main reason for this depressing situation is the unavailability of rapid, more sensitive and specific techniques for the timely diagnosis of TB. The early and accurate identification of MTB facilitates control, prevention and treatment of this chronic disease. In addition TB detection and treatment are also difficult in underdeveloped countries, like Pakistan, that are facing multifaceted emergencies, including humanitarian crises and conflicts [14]. Accurate and timely diagnosis is crucial for timely treatment that might minimize the risk of disease transmission. Several techniques are available for the diagnosis of TB such as smear examination, culturing, ELISA based and PCR-based detection methods. Among these culturing of the *M. tuberculosis *remains the gold standard method for diagnosing TB however it is time consuming and takes 5-6 weeks. Direct smear examination under microscope is the most popular among all the methods currently employed worldwide for TB diagnosis as it is very rapid but has the issues of low sensitivities and low specificities. In view of the huge convenience of smear microscopy, frequent efforts have been made to improve its sensitivity and specificity [16-18], however, the smear examination method still has the sensitivity problem as direct smear microscopy lacks sensitivity and versatility in terms of application to extra-pulmonary specimens. After the discovery of polymerase chain reaction (PCR) technique, maximum attention has been given to developing nucleic acid based amplification diagnostic techniques due to its speed, specificity and sensitivity. Several TB gene targets based on automated and semi-automated kits for *mycobacterial *DNA detection are available and are being extensively evaluated [19-22].

In the current study we studied the clinical utility of MTB PCR test for the rapid diagnosis of both extra-pulmonary and pulmonary tuberculosis with higher sensitivity in a variety of clinical samples such as whole blood, serum/plasma, CSF, Ascetic fluid, Urine, Sputum, Bronchoalveolar Lavage (BAL), Pleural fluid, Pericardial fluid, Pus and Bone marrow. We were able to detect TB DNA in 46.5% (356/766) smear negative TB samples by PCR amplification method. It has previously been estimated that the sensitivity and specificity rates for *M. tuberculosis *culture that is a reference standard are approximately 96% and 81%, however their significant variability in different studies limits their value for clinical diagnostic purposes [23]. For example most recently the reported positivity rate of TB is just 15%-20% in Pakistan using culturing on a very large number of (more than 50,000) specimen received from different geographical areas of the country [24] that is very low.

The positivity rates were different in different types of pulmonary and extra-pulmonary TB samples. It was observed that 70% of the BAL samples were found TB positive followed by 67.6% TB positive CSF samples though all these were smear negative. In pericardial TB samples the positivity rate was 30%. Additionally this method can detect TB with same high sensitivity in males and females as no significant difference of TB positivity in males (46.4%) and females (46.5%) were seen. Regarding positivity rates in different specimens, among males, higher positivity rates were observed in blood, ascetic fluid, pericardial fluid, pus and BAL samples while females showed higher positivity rates in urine, CSF, Pleural fluid, bone marrow and sputum samples. Effect of age on samples positivity was also being evaluated. It was found that most of the positive samples lie in the age group of 41 to 60 years (35.6%) while 34.2% samples were positive in the ages between 21 years and 40 years.

With respect to the diagnostic value of PCR in the evaluation of patients with suspected pulmonary and extra-pulmonary TB, we observed that, because of its very high sensitivity with AFB smear negative samples, the test can be confidently used to accurately diagnose TB. Patients having TB like symptoms with negative PCR might be due to opportunistic infectious agents particularly due to non *tuberculos mycobacterium *(NTM). The propensity of both clinical suspicion and positive TB PCR to overestimate the probability of TB might lead to mistaken initiation of therapy, isolation, and contact investigation (23).

## Conclusion

We conclude that PCR assay is highly sensitive and specific tool available to date for the diagnosis of *M tuberculosis *in all types of specimens obtained from patients with a clinical suspicion of tuberculosis whether pulmonary or extra-pulmonary and can be reliably used for rapid identification of TB.

## Abbreviations

TB: *tuberculosis*; PCR: polymerase chain reaction; MTB: *Mycobacterium tuberculosis*; CSF: cerebro spinal fluid; BAL: bronchoalveolar lavage; MDR: multiple drug resistance; XDR: extensive drug resistance; WHO: World Health Organization.

## Competing interests

The authors declare that they have no competing interests.

## Authors' contributions

IA, ZA, MA, MS and SA conducted all the experiments. MI and IA reviewed the literature and wrote the manuscript. MI guided conducting the whole experiment and edited the manuscript. AH helped in literature review. All the authors read and approved the final manuscript.
